# DEVELOPING A CONCEPTUAL MODEL OF FAMILY PREPAREDNESS FOR FUTURE DEMENTIA CAREGIVING IN CHINESE FAMILIES

**DOI:** 10.1093/geroni/igz038.1744

**Published:** 2019-11-08

**Authors:** Jacky C P Choy

**Affiliations:** Department of Social Work and Social Administration, The University of Hong Kong, Hong Kong, Hong Kong

## Abstract

Dementia is a growing health challenge that demands better public preparedness. Persons with dementia often lack the capacity to make and execute plans such that family involvement in care preparation becomes necessary. It is commonly observed in Chinese societies that there are more than one family members involved in the taking care of the person with dementia. The current qualitative study aims to understand preparedness for dementia caregiving of a family as unit in a Chinese society. In-depth interviews with 10 family units of dementia caregivers were conducted. Participants (4 spousal caregivers; 44 to 80 years old; mean years of caregiving: 3.3) reflected on how prepared their families were before the caregiving began. Thematic analysis was applied to examine the family preparedness and the family dynamics throughout the caregiving journey. As opposed to a crisis-driven involvement, involvement of more family members before crisis was helpful for reducing the damage brought to the family. Furthermore, families that could align their expectation and understanding of the situation, share knowledge and resources, negotiate the allocation of caregiving duties, and provide emotional support among family members were more likely to provide proper care with minimal sacrifice in family wellbeing. Chinese families often worked as a caregiving team, yet, with uneven distribution of caregiving duties and a lack of proper communication to sustain their caregiving role healthily. Timing and quality of family involvement were more influential factors than family resources to successful adaptation to caregiving.

